# Soil-Transmitted Helminthiasis in Children from a Rural Community Taking Part in a Periodic Deworming Program in the Peruvian Amazon

**DOI:** 10.4269/ajtmh.18-1011

**Published:** 2019-07-15

**Authors:** Renato A. Errea, George Vasquez-Rios, Maria L. Calderon, Diego Siu, Kevin R. Duque, Luciana H. Juarez, Rodrigo Gallegos, Celene Uriol, Claudia R. Rondon, Katia P. Baca, Rosario J. Fabian, Marco Canales, Angelica Terashima, Luis A. Marcos, Frine Samalvides

**Affiliations:** 1 Instituto de Medicina Tropical “Alexander von Humboldt,” Universidad Peruana Cayetano Heredia, Lima, Peru;; 2 Department of Global Health and Social Medicine, Harvard Medical School, Boston, Massachusetts;; 3 Department of Internal Medicine, Saint Louis University School of Medicine, Saint Louis, Missouri;; 4 Facultad de Medicina Alberto Hurtado, Universidad Peruana Cayetano Heredia, Lima, Peru;; 5 Scientific Society of Medical Students, Universidad Peruana Cayetano Heredia, Lima, Peru;; 6 Infectious Diseases Division, SUNY/Stony Brook University, Stony Brook, New York;; 7 Department of Infectious, Tropical and Dermatological Diseases, Hospital Cayetano Heredia, Lima, Peru

## Abstract

Children in the Peruvian Amazon Basin are at risk of soil-transmitted helminths (STH) infections. This study aimed to determine the prevalence of STH infection in children from a rural Amazonian community of Peru and to elucidate epidemiological risk factors associated with its perpetuation while on a school-based deworming program with mebendazole. Stool samples of children aged 2–14 years and their mothers were analyzed through direct smear analysis, Kato–Katz, spontaneous sedimentation in tube, Baermann’s method, and agar plate culture. A questionnaire was administered to collect epidemiological information of interest. Among 124 children, 25.8% had one or more STH. Individual prevalence rates were as follows: *Ascaris lumbricoides*, 16.1%; *Strongyloides stercoralis*, 10.5%; hookworm, 1.6%; and *Trichuris trichiura*, (1.6%). The prevalence of common STH (*A. lumbricoides*, *T. trichiura*, and hookworm) was higher among children aged 2–5 years than older children (31.6% versus 12.8%; *P* = 0.01). In terms of sanitation deficits, walking barefoot was significantly associated with STH infection (OR = 3.28; CI 95% = 1.11–12.07). Furthermore, STH-infected children more frequently had a mother who was concomitantly infected by STH than the non-STH–infected counterpart (36.4% versus 14.1%, *P* = 0.02). In conclusion, STH infection is highly prevalent in children from this Amazonian community despite routine deworming. Institutional health policies may include hygiene and sanitation improvements and screening/deworming of mothers to limit the dissemination of STH. Further studies are needed to address the social and epidemiological mechanics perpetuating these infections.

## INTRODUCTION

Infections by the soil-transmitted helminths (STH), including *Ascaris lumbricoides*, *Trichuris trichiura*, *Ancylostoma duodenale*/*Necator americanus* (hookworms), and *Strongyloides stercoralis*, disproportionately affect children around the world.^1^ Because of their transmission associated with poor sanitary conditions and inadequate hygiene practices, higher burden of disease is seen in children from developing countries from sub-Saharan Africa, Southeast Asia, and Latin America.^1^ Approximately 267 million preschool-age children (PSAC) and 568 million school-age children (SAC) worldwide are at risk of STH infection as well as impaired child growth and cognitive development from *A. lumbricoides*, *T. trichiura*, and hookworm infections,^2^ and death due to severe *S. stercoralis* infection.^3^ Thus, their control is a global health priority.

Water, sanitation, and hygiene (WASH) interventions alongside repeated chemotherapy at regular intervals are tenets for the control of STH.^4,5^ Despite the progress made, there are still 49% PSAC and 31% SAC in endemic countries not receiving preventive chemotherapy.^6^ Similarly, 2.3 billion people do not have adequate sanitation service, 892 million practice open defecation, and 884 million lack clean drinking water.^7^ Higher scale of deworming programs in endemic countries and global outreach of WASH programs are necessary.

The Amazon Basin in Peru is at special risk of STH infection because of its weather condition (favoring geohelminth survival) and the health inequalities present in the region. In Peru, although health interventions have rapidly increased in poor areas of the Andes, the Amazon has experienced less and slower health progress.^8^ Moreover, some antihelminth programs already implemented in the region have reported low chemotherapy drug coverage.^9^ Further research in Amazonian communities are needed to comprehensively identify barriers for the control of STH. The aim of this study was to determine the prevalence of STH infection in children in a rural community in the Amazon jungle, as well as to identify demographic, sanitation, and clinical factors associated with these parasitic infections.

## METHODS

### Study design.

We conducted a cross-sectional study to determine the point prevalence of *A. lumbricoides*, *T. trichiura*, hookworm, and *S. stercoralis* infections in children aged 2–14 years from Padre Cocha, an Amazonian community in Peru, through December 2nd–13th, 2015. This study included multiple stool-based diagnostic techniques to increase parasite detection, including direct smear examination, Kato–Katz technique (K-K), spontaneous sedimentation in tube technique (SSTT), modified Baermann technique (MBT), and agar plate culture (APC). Information about children’s demographics, past medical history, hygiene practices, and household sanitation conditions was obtained. Also, we explored the burden of STH in mothers of the participating children and examined the association between maternal characteristics and children’s STH infection status.

### Study population.

**Figure 1.**
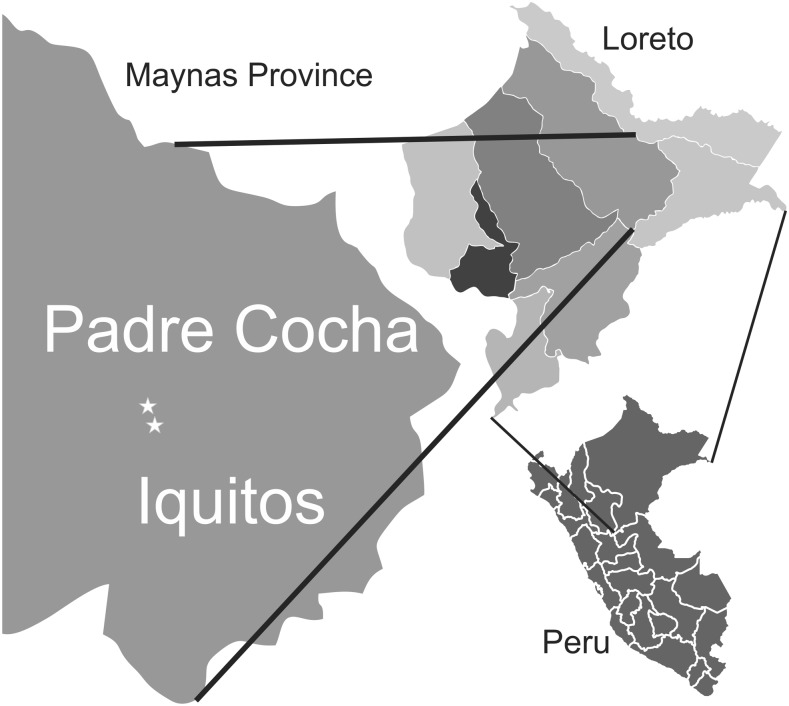
Location of Padre Cocha.

The community of Padre Cocha is part of the district of Punchana, province of Maynas, in the region of Loreto, Peru, and it is located in the bay of the Nanay River (one of the main tributaries of the Amazon River) (Figure 1). It is only accessible by river and located 20 minutes away from the closest urban area, Iquitos city. Its population comprises 850 inhabitants, distributed in 195 households.^10^ In 2012, approximately 31% of the habitants were considered poor and 11% lived in extreme poverty. The main economic activities include fishing, farming, and craft-making. The great majority of inhabitants speak Spanish as their mother language.

Since 2012, a school-based deworming program was initiated in the region of Loreto. The program consisted on the administration of 500 mg of mebendazole on a quarterly basis to children aged 3–17 years, aiming to reduce the burden of *A. lumbricoides*, *T. trichiura*, and hookworm infections (known as common STH).^9^ The deworming program had been implemented for almost 4 years by the time of this study. Drugs were provided both in the nursing school to children aged 3–5 years, as well as in the elementary, middle, and high schools for children aged 6–17 years.^11^

### Data and stool collection.

Authorization and support from the community leaders were obtained. Households with eligible children were identified: children aged 2–14 years and residing in Padre Cocha for at least 1 year. After obtaining informed consent from parents, children were approached at the local school or in their households and only those who assented to participate were included. Mothers who were willing to participate provided informed consent as well. A questionnaire was administered to collect information on sociodemographic characteristics, relevant medical history, hygiene practices, and household sanitation for both children and mothers. Weight and height of children were measured using calibrated instruments.

Two labeled plastic containers without preservatives were given to each participant to collect the stools. Mothers were instructed about appropriate stool sample collection techniques. Each stool sample was obtained at least 24 hours apart and was immediately transported to the local health center. The research team collected the stool samples every 6–12 hours at the local health center and rapidly transported them for analysis in Iquitos city.

### Parasitological analyses.

Stool samples were evaluated in Iquitos city immediately on reception. Senior laboratory professionals from the Institute of Tropical Medicine Alexander von Humboldt-Universidad Peruana Cayetano Heredia, analyzed the samples. The first part of the analysis consisted in a macroscopic analysis for stool consistency, presence of blood, mucus, or adult helminth parasites. During the microscopic analysis, five stool-based parasitological techniques were conducted, including direct smear examination, SSTT, K-K, MBT, and APC. Spontaneous sedimentation in tube technique and K-K were performed mainly for the detection of *A. lumbricoides*, *T. trichiura*, and hookworm infection,^12^ whereas MBT and APC were used for the detection of *S. stercoralis*.^13^ We considered a positive stool sample if the parasite was detected by any of these diagnostic methods. Parasitological techniques were performed following the guidelines for intestinal parasites diagnosis from the National Institute of Health of Peru.^14^

### Sample size calculation and statistical analysis.

From a total of 850 inhabitants in Padre Cocha, approximately 32% of them are younger than 15 years.^10,15^ Based on previous epidemiological reports, the expected frequency of any STH was 50%, as of December 2015.^9^ Thus, the estimated sample considering a 5% margin of error was 136, as per Epi Info 3.5.4 (CDC, Atlanta, GA). The point prevalence of STH infection was defined as the prevalence of one or more STH in the stool samples; individual prevalence rates were also calculated for each parasite. Children aged 2–5 years were considered as PSAC and those aged between 6 and 14 years were considered as SAC. Data were entered in a Microsoft Excel spreadsheet and posteriorly evaluated using STATA v14 (StataCorp, College Station, TX). Categorical data were evaluated by χ^2^ test or Fisher’s exact test with a significance level of 0.05. Bivariate analyses with odds ratio calculation were conducted to assess the association between STH infection and the characteristics of interest considering a 95% CI. Missing data were reported when values missing were > 5%.

### Ethics statement.

This study was approved by the Institutional Review Boards of Universidad Peruana Cayetano Heredia (IRB #65959) and Hospital Cayetano Heredia (IRB #126-015). Parents provided written informed consent for participating children. Children who participated provided their assent. Mothers who were also willing to participate provided written informed consent as well.

## RESULTS

Stool samples from 124 children were collected. The mean age was 7 ± 3.4 years, and 53.2% were male. In terms of age classification, 30.7% were PSAC, whereas 69.3% were SAC. Regarding anthropometric characteristics, 60.8% had stunting based on the WHO criteria. During the last year, 79% of the children took antiparasitic medicines, 23% reported at least three episodes of diarrhea, and 14.6% were significantly absent from school (≥ 4 missed days). About hygiene practices, 84% reported inconsistent handwashing, 66.7% walked barefoot, 28.2% were nail biters, and 20.1% used the river for bathing or recreational activities. Children included in the study belonged to 78 households characterized by the lack of running water (55.6%) and of pavemented floor (40.8%); 97.4% had latrines for defecation.

The point prevalence of STH infection among children was 25.8%. *Ascaris lumbricoides* was the most frequent parasite (16.1%), followed by *S. stercoralis* (10.5%); the prevalence of hookworm and *T. trichiura* infections was the same (1.6%) (Table 1). The point prevalence of STH tended to be higher among PSAC than among SAC (36.8% versus 20.9%; *P* = 0.06). When considered only the most common STH (*A. lumbricoides*, *T. trichiura*, and hookworm), the prevalence was significantly higher in PSAC than in SAC (31.6% versus 12.8%; *P* = 0.01). During the assessment of factors associated with STH infection, walking barefoot was a risk factor for the infection with STH (OR = 3.28; 95% CI = [1.11–12.07]; *P* = 0.02). Further details are seen in Table 2.

**Table 1:** Prevalence of soil-transmitted helminth infection in 124 children in Padre Cocha

	No	%
*Ascaris lumbricoides*	20	16.1
*Strongyloides stercoralis*	13	10.5
*Necator americanus/Ancylostoma duodenale*	2	1.6
*Trichuris trichiura*	2	1.6
Any STH	32	25.8

STH = soil-transmitted helminth.

**Table 2:** Association between soil-transmitted helminth infection and past medical history, hygiene practices, and household sanitation characteristics in children in Padre Cocha

Characteristic	STH infection (*n* = 32) No (%)	No STH infection (*n* = 92) No (%)	OR	95% CI	*P*-value
≥ 3 episodes of diarrhea in the last year	8/32 (25)	23/91 (25.3)	0.99	0.34–2.68	0.98
Chronic malnutrition	19/31 (61.3)	54/89 (60.7)	1.03	0.41–2.63	0.95
≥ 4 days school absenteeism in the last year	5/31 (16.1)	13/91 (14.3)	1.15	0.29–3.88	0.78
Walking barefoot	26/31 (83.9)	56/92 (60.9)	3.28	1.11–12.07	0.02
Irregular handwashing	24/32 (75)	80/92 (87)	0.45	0.15–1.44	0.11
Use of river	12/31 (38.7)	31/92 (33.7)	1.24	0.48–3.11	0.61
Nail biting	7/32 (21.9)	28/92 (30.4)	0.64	0.21–1.76	0.35
Soil floor	15/32 (46.9)	36/91 (39.6)	1.35	0.55–3.28	0.47
Open defecation	0/32 (0)	1/90 (1.1)	–	–	–
No running water	16/31 (51.6)	51/88 (58)	0.77	0.31–1.92	0.54

STH = soil-transmitted helminth.

Stool samples from 51 mothers were also collected, representing the caregivers of 69.4% of the pediatric population that participated in this study. The mean age was 35.4 ± 9.2 years. The point prevalence of STH infection in this subgroup was 19.6%: 9.8% were infected by *A. lumbricoides*, 5.9% by *S. stercoralis*, and 3.9% by hookworms; none were infected by *T. trichiura*. Twenty-five percent were single mothers and 59.2% had low education level (incomplete high-school education or below). There was no significant statistical difference between single marital status, low educational level, or young age (≤ 25 years) of the mother and STH infection in children (Table 3). However, children who were infected by STH were more likely to have a mother who was also infected by one of these organisms when compared with non-STH–infected children (36.4% versus 14.1%, *P* = 0.02).

**Table 3:** Association between characteristics of the mothers and soil-transmitted helminth infection in children in Padre Cocha

Characteristic	STH infection in children (*n* = 22), No (%)	No STH infection in children (*n* = 64), No (%)	*P*-value
Single marital status	5/22 (22.7)	16/64 (25)	0.83
Young age	4/22 (18.2)	4/63 (6.4)	0.12
Low educational status	17/22 (77.3)	36/62 (58.1)	0.11
STH infection	8/22 (36.4)	9/64 (14.1)	0.02

STH = soil-transmitted helminth.

## DISCUSSION

A high overall prevalence of STH infection (26%) was found among children from an Amazonian rural community who were part of a school-based deworming program. Most of the burden was related to *A. lumbricoides* infection (16.5%) despite its known susceptibility to mebendazole.^16^ Also, a relatively high prevalence of *S. Stercoralis* (10.5%) was noticed, which is concordant to that reported in a local study.^17^ By contrast, infections by *T. trichiura* and hookworm were rare in this study when compared with other studies conducted in the Peruvian rainforest.^9,12,18,19^

The prevalence of the common STH (*A. lumbricoides*, *T. trichiura*, and hookworm) was significantly higher among PSAC than among SAC (31.6% versus 12.8%, *P* = 0.01). This finding is important as recent evidence has shown robust positive health effects of deworming in PSAC, reflected in decreased stunting and anemia rates.^20^ In Padre Cocha, PSAC and SAC attended separate schools and were approached by the deworming program staff under different procedures. This may have resulted in different practices, medication supply, or program surveillance between PSAC and SAC.^21^

*Strongyloides stercoralis* infection was common in children of this community. Strongyloidiasis can cause diarrhea and abdominal pain in the pediatric population and has also been associated with stunting in children with severe infection.^22,23^ Mebendazole, the drug administered as part of the deworming program in this community, is not effective against *S. stercoralis*.^16^ On the other side, thiabendazole and ivermectin have shown to be effective within strongyloidiasis control programs elsewhere.^24–26^ Appropriate strategies including therapy with thiabendazole or ivermectin could potentially contribute to reduce the extent of infection among children. Unfortunately, and to the best of our knowledge, there are presently no national policies that promote the eradication of *S. stercoralis* in children or adults in Peru.

A great proportion of participants lacked optimal sanitation conditions and adequate hygiene practices. Although several risk factors were assessed, walking barefoot was the only characteristic that was significantly associated with a higher STH burden. The lack of power of this study may have limited the elucidation of further factors associated with parasite infection as inconsistent hygiene practices, lack of clean water, or pavemented floor have been described as important factors that may increase the rate of STH infection.^4,27^ Interventions that promote hygiene education have been found to be effective in reducing helminth infection in other local Amazonian communities.^19^ Padre Cocha urges similar education initiatives as well as sustained public efforts for improving access to clean water and pavement floor as key mechanisms to tackle these infectious diseases.

More than half of the pediatric population studied had stunting, proportion substantially higher than the 17.3% national estimate.^8^ Although stunting was not associated with STH infection in this study, also probably because of underpower, several studies have reported the effect of moderate-to-high STH infection rates on child nutritional status.^28^ Moreover, low nutritional status also influences the impact of STH infections.^28^ As has also been found by neighbor countries,^29^ rural Amazon communities may be at special risk of children stunting. Thus, nutrition status of children in Padre Cocha requires public health attention.

In this study, children who were infected by STH were more likely to have a mother who was also infected by one of these organisms when compared with non-STH–infected children. Helminth infection in mothers has been reported as a risk factor for infection in children and could potentially contribute to the high prevalence of STH infection in this population because they likely share the same source of the infection.^30^ As mothers are not part of the MDA local program, infected mothers could be playing a role in the perpetuation of STH infection within the household. Following the present WHO recommendation of also administering antiparasitic drugs to women of childbearing age,^5^ mass administration in mothers may help to control STH infection in this community. Similarly, although not assessed in this study, other household members could also be infected; thus, it may also be appropriate to study the impact of expanding the deworming program not only to mothers but also to the entire community, as a meta-analysis has shown better morbidity outcomes of community-wide programs than selective approaches.^31^ This could contribute to elucidate the best effective strategy in this community.

The study had several limitations. First, accurate information about previous participation in the deworming program was not available, which impeded to assess the relationship between recent antihelminthic drug administration and STH infection. Second, the lack of multiple stool samples per participant could have reduced the detection rate of parasites; however, we used multiple parasitological techniques per sample to increase the rate.^12,13^ Also, for the specific case of *S. stercoralis*, the use of serology-based tests could have increased its detection because of their reported higher sensitivity; however, concerns about specificity as well as the challenge of implementing such tests under limited laboratory conditions of on-field research studies remain.^32^ Fourth, our results are underpowered because of the relatively small sample size. This also prevented further multivariate analysis of meaningful risk factors. Fifth, we only obtained stool samples from around 70% of the mothers. In addition, mothers from infected children could have been more motivated to participate than mothers from uninfected children, representing a potential selection bias. Despite these limitations, our study may represent the context of other similar rural communities in the Peruvian rainforest and our results should be discussed to promote alternative approaches to the control of STH.

In conclusion, STH infection was highly prevalent in children in Padre Cocha despite routine deworming. Among helminth infections, *A. lumbricoides* and *S. stercoralis* were the most frequent. Preschool-age children and children who walk barefoot may be at an increased risk of STH burden. Future studies may address the relationship between maternal and children infection. Improvement of sanitary and hygiene conditions is also necessary in this community.
